# Anatomical and Functional Differences in the Sex-Shared Neurons of the Nematode *C. elegans*

**DOI:** 10.3389/fnana.2022.906090

**Published:** 2022-05-06

**Authors:** Dongyoung Kim, Byunghyuk Kim

**Affiliations:** Department of Life Science, Dongguk University-Seoul, Goyang, South Korea

**Keywords:** sex difference, sexual dimorphism, nervous system, neurite branching, synaptic connectivity, functional modulation, neural cell-surface protein, *C. elegans*

## Abstract

Studies on sexual dimorphism in the structure and function of the nervous system have been pivotal to understanding sex differences in behavior. Such studies, especially on invertebrates, have shown the importance of neurons specific to one sex (sex-specific neurons) in shaping sexually dimorphic neural circuits. Nevertheless, recent studies using the nematode *C. elegans* have revealed that the common neurons that exist in both sexes (sex-shared neurons) also play significant roles in generating sex differences in the structure and function of neural circuits. Here, we review the anatomical and functional differences in the sex-shared neurons of *C. elegans*. These sexually dimorphic characteristics include morphological differences in neurite projection or branching patterns with substantial changes in synaptic connectivity, differences in synaptic connections without obvious structural changes, and functional modulation in neural circuits with no or minimal synaptic connectivity changes. We also cover underlying molecular mechanisms whereby these sex-shared neurons contribute to the establishment of sexually dimorphic circuits during development and function differently between the sexes.

## Introduction

Sexually reproducing animals often show innate, stereotyped sexual behaviors for mating. These innate behaviors are differently displayed in the two sexes and result from the sexual dimorphism in the structure and function of the nervous system (Knoedler and Shah, 2018; McKinsey et al., 2018; Goodwin and Hobert, 2021). To what extent the nervous system differs between the sexes and how the sexually dimorphic neural structure is generated during development have been interesting topics in the field of the neurobiology of sex.

Previous studies that have mainly used invertebrate model organisms have revealed numerous examples of sexual dimorphism in the nervous system and described how these dimorphic structures emerge through sexual differentiation programs employing key transcription factors. In the fruit fly *Drosophila melanogaster*, for example, two transcription factors acting in the sex-determination pathway, Fruitless (Fru) and Doublesex (Dsx), are responsible for the sexual differentiation of the brain between males and females (Billeter et al., 2006). The nematode worm *Caenorhabditis elegans* has two sexes, hermaphrodites and males, and the transcription factor TRA-1 in the sex-determination pathway acts as a master regulator of the sexual characteristics of nearly all the somatic cells, including neurons (Hodgkin, 1987; Portman, 2007). In hermaphrodite worms, TRA-1 represses male-specific genes, thereby inducing hermaphrodite-specific characteristics. Conversely, in males, TRA-1 is inactivated, and thus male-specific genes are expressed, generating male-specific characteristics (Hunter and Wood, 1990; Schvarzstein and Spence, 2006). As a consequence of TRA-1 regulation, numerous sexually dimorphic characteristics appear during sexual maturation in *C. elegans*, including differences in morphology, gene expression, and neuronal structure and function (Sulston et al., 1980; Kim et al., 2016; Barr et al., 2018).

The *C. elegans* nervous system contains 294 neurons that are shared in both sexes (sex-shared neurons) in addition to the neurons specific to each sex (sex-specific neurons)—eight in hermaphrodites and 93 in males (Sulston et al., 1980; Barr et al., 2018; Molina-García et al., 2020). Although the sex differences in neural circuits largely result from the dimorphic wiring patterns contributed by sex-specific neurons, recent studies have revealed that sex-shared neurons also play significant roles in generating sex differences in the structure and function of neural circuits. In this review, we summarize the known morphological and functional differences in the sex-shared neurons of *C. elegans*. We discuss the molecular mechanisms whereby these sex differences in shared neurons contribute to the establishment of sexually dimorphic circuits, especially by focusing on the roles of transcription factors and the corresponding effectors, including neural cell-surface proteins.

## Morphological Differences

**Figure 1:**
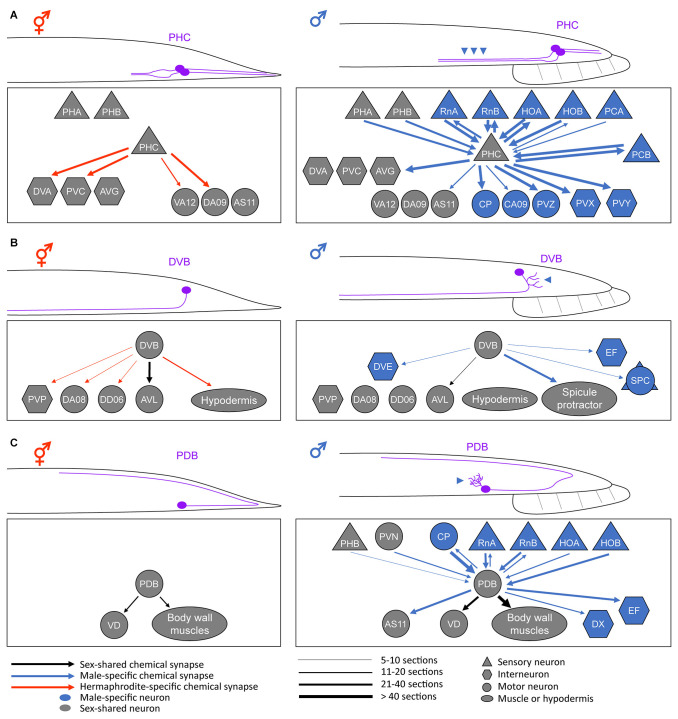
Anatomical sex differences in the sex-shared neurons. Schematic diagrams of the sexually dimorphic neurite morphology and synaptic connectivity in a pair of PHC neurons **(A)**, DVB neuron **(B)**, and PDB neuron **(C)**. For simplicity, only the posterior half of a worm is drawn. Neurite or neurite branches specifically elongated in males are indicated by the arrowheads. For the connectivity, the synaptic weights of the chemical synapses (calculated based on the numbers of electron-micrograph sections) are depicted based on Cook et al. (2019). Note that the sex differences in neurite morphology largely affect the neural connectivity pattern, thereby resulting in different circuit functions (see text). Adapted from Serrano-Saiz et al. (2017a); Hart and Hobert (2018); and Pereira et al. (2019).

Obvious sex differences in the sex-shared neurons of *C. elegans* can be found in the patterns of neurite projections or branches. As described below, in most cases, males have additional neurite branches in comparison to hermaphrodites, thereby generating additional synaptic connections with other partner neurons or the muscle tissue. Accordingly, the shared neurons in males often acquire functional properties different from those in hermaphrodites. In this section, we will describe examples of such morphological differences, accompanying changes in synaptic connectivity and neuronal functions, and molecular mechanisms underlying these sexually dimorphic characteristics. The sexual differences in neurite pattern and synaptic connectivity are depicted in Figure 1. Beyond our discussion, there are also two male-specific neurons—MCM and PHD—which originate from glial cells shared by both sexes but are specified into neurons only in males, and the relevant information is provided elsewhere (Sammut et al., 2015; Molina-García et al., 2020).

### PHC Neurons

Any changes in the neurite projection pattern can affect the connectivity and function of a neuron. While some of such modifications can form throughout development in *C. elegans* (Witvliet et al., 2021), some result from the sexual differentiation of sex-shared neurons, such as PHC (Serrano-Saiz et al., 2017a). The *C. elegans* nervous system contains a pair of PHC neurons located in the tail (Altun and Hall, 2011). They show strong sexual dimorphism in neurite projection length; the male PHC axon is approximately two times longer than the hermaphrodite PHC axon (Figure 1A). At the last larval stage, when most of the sexual differentiation processes occur, PHC begins to extend its axon only in males (Serrano-Saiz et al., 2017a). This male-specific PHC axon extension results in the synapse formation between PHC and many male-specific neurons as well as other sex-shared neurons (Jarrell et al., 2012; Cook et al., 2019; Figure 1A).

The sexual differences in PHC axon length and the resulting synaptic connectivity manifest as sexually dimorphic PHC functions. The hermaphrodite PHC has few synaptic inputs but many outputs into a set of interneurons and functions as a sensory neuron for nociception (e.g., harsh touch) and temperature sensation (White et al., 1986; Liu et al., 2012; Serrano-Saiz et al., 2017a). By contrast, the male PHC, with many more synaptic connections than the hermaphrodite counterpart, acts as a hub neuron required for vulva location behavior, a specific step in male mating behavior (Jarrell et al., 2012; Serrano-Saiz et al., 2017a). In parallel to the morphological PHC remodeling, the expression of several synaptic vesicle components and a neuropeptide are upregulated in the male PHC, supporting the functional differentiation of PHC during sexual maturation (Serrano-Saiz et al., 2017a).

The above-mentioned sexually dimorphic PHC characteristics, namely different axon lengths and differential expression of the neuropeptide and synaptic factors, are under the control of the master regulator TRA-1 in the sex-determination pathway. It has been shown that the transcription factor Double sex/MAB-3 domain protein DMD-3 is necessary and sufficient for sex-specific PHC differentiation. DMD-3 is expressed in PHC, and this process is regulated transcriptionally and cell autonomously by TRA-1 (Serrano-Saiz et al., 2017a). To date, the effector molecules downstream of DMD-3 or TRA-1, which induce the male-specific PHC axon extension, have not been identified. However, the axon extension is likely mediated by neural cell-surface proteins since these proteins are involved in multiple, conserved steps of neural circuit assembly, such as axon guidance, fasciculation, and targeting (De Wit and Ghosh, 2016; Kim, 2019; Jin and Kim, 2020). Indeed, an adhesion type of neural cell-surface protein called SAX-7 (the *C. elegans* ortholog of L1CAM) has been shown to be expressed in the male PHC and required for the axon fasciculation with other neurons in male *C. elegans* (Kim and Emmons, 2017).

### DVB Neuron

DVB is a GABAergic motor/interneuron located in the tail of *C. elegans*. The DVB neurite is not branched in hermaphrodites but extends numerous branches toward the posterior direction in males (Figure 1B). These male-specific extra branches are formed in adulthood and make new synapses with a subset of male-specific neurons and muscles that control a male copulatory structure called spicule (Figure 1B). Functionally, DVB controls defecation behavior in both sexes but also promotes spicule protraction for mating in males, which is consistent with the changes in neural connectivity (LeBoeuf and Garcia, 2017; Cook et al., 2019).

Interestingly, the male-specific DVB neurite outgrowth appears to be dependent on experience but not on the sex-determination pathway. DVB neurite length increases throughout adulthood. The neurites grow normally if the male is allowed to mate but do not grow if the male is prevented from mating. The optogenetic activation of DVB postsynaptic target cells induces neurite outgrowth, indicating that DVB neurite outgrowth is greatly influenced by mating experience (Hart and Hobert, 2018). However, the DVB neurite structure was not altered upon changes in the sexual identity of DVB or its targets (by genetically modulating TRA-1 activity in these cells), suggesting no relevance of the sex-determination pathway in DVB neurite outgrowth (Hart and Hobert, 2018).

Molecular mechanisms that regulate the DVB neurite plasticity in males involve two neuronal cell-surface proteins. Neurexin, a well-known synaptic adhesion protein, and its trans-synaptic partner neuroligin have been shown to contribute to the formation of extra DVB branches in males (Hart and Hobert, 2018). NRX-1 (the *C. elegans* ortholog of neurexin) promotes DVB neurite branching in a cell-autonomous manner, whereas NLG-1 (the ortholog of neuroligin), expressed in the postsynaptic DVB target cells suppresses the effect of NRX-1. The antagonistic functions of NRX-1 and NLG-1 are not likely involved in their canonical functions in synapse formation (Südhof, 2017; Hart and Hobert, 2018). How the same interacting pair of cell-surface proteins separately function in neurite branching and synapse formation remains to be investigated.

### PDB Neuron

PDB is a motor neuron located in the tail region of both sexes. PDB also exhibits a sexually dimorphic feature; it has elaborate neurite branches in males but not in hermaphrodites (Figure 1C). Before sexual maturation, the PDB neurite remains unbranched; however, the male PDB forms extensive neurite branches during the transition from the juvenile stage to the adult stage (Pereira et al., 2019). These male-specific branches receive numerous synaptic inputs, mainly from a group of male-specific neurons (Jarrell et al., 2012; Cook et al., 2019; Figure 1C). Therefore, it is expected that sexually dimorphic features in behavior can arise due to the connectivity changes in PDB. Ablation of PDB showed that this neuron is required for locomotion in hermaphrodites, especially for the control of the ventral-dorsal movement of the body (Yan et al., 2017). Based on its synaptic connectivity in males, PDB has been postulated to control the ventrally arched posture of the male during mating (Emmons, 2014). Thus, further studies are needed to test the hypothesis on the behavioral outcomes of sexually dimorphic PDB function.

The sexually dimorphic PDB branching seems to be regulated by the timing of sexual maturation as well as the sex-determination pathway. Males mutant for the transcription factor LIN-29, a downstream effector of the core *lin-28/let-7/lin-41* heterochronic pathway, lack the male-specific PDB branching and show an abnormal male mating behavior (Pereira et al., 2019). Although the neuronal expression of LIN-29 has been shown to be controlled by TRA-1 (Pereira et al., 2019), the branching factors (most likely cell-surface proteins) involved in the male-specific PDB branch formation and the mechanisms of their regulation have not been identified yet.

### Other Neurons

Reconstructions of the *C. elegans* nervous system *via* electron microscopy have revealed that some other sex-shared neurons also show morphological sex differences. These cells include the interneurons AVG and AVF and the motor neuron DD06, which show male-specific neurite branching with sexually dimorphic connectivity. These additional branches are formed at the caudal end of males and receive synaptic inputs from male-specific neurons (Cook et al., 2019; skeleton neuron diagrams and connectivity are available in WormWiring^1^). *Via* fluorescence microscopy, at least AVG has been shown to form a male-specific branching pattern (Kim and Emmons, 2017). Future studies should assess the other neurons for additional branches in males and examine the ensuing functional differences caused by these changes as well as the underlying molecular mechanisms.

## Differences in Synaptic Connectivity

A comparison of the connectomes of male and hermaphrodite *C. elegans* showed that up to 30% of the synaptic connections among the sex-shared neurons differ between the sexes (Cook et al., 2019). Most of these differences result from sexually dimorphic choices of synaptic partners, without any obvious sex difference in neuronal structure, although a few cases are due to the connectivity changes accompanied by structural changes as described above. The differences in neural connectivity between the two sexes, in which connections are present or abundant only in one sex, have been confirmed by visualizing the synaptic connections *via* the GRASP (GFP reconstitution across synaptic partners) or iBLINC (*in vivo* biotin labeling of intercellular contacts) technique (Feinberg et al., 2008; Desbois et al., 2015). These cases include specific or abundant synaptic connections in males (e.g., PHB > AVG, AVG > VD13, AVG > DA09, RIA > RIB, ASI > AFD, IL2 > RIB, and IL1 > RIB) and in hermaphrodites (e.g., PHB > AVA, PHA > AVG, ADL > AVA, and ASH > AVA; Oren-Suissa et al., 2016; Cook et al., 2019). How these connections are sexually regulated is less understood except for the PHB > AVG and PHB > AVA connections and their generation mechanisms.

**Figure 2:**
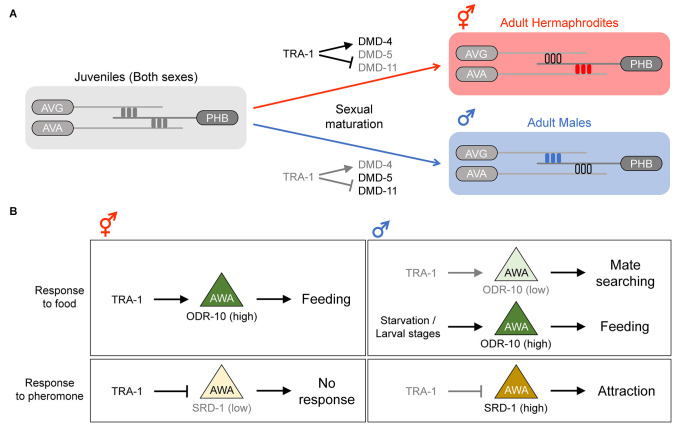
Sex differences in synaptic connectivity and function of the sex-shared neurons. **(A)** Generation of sex-specific synaptic connections during the development. In larval stages, both sexes have identical synaptic connections. In the adult stage, sex-specific pruning events during sexual maturation result in sexually dimorphic connectivity; the PHB > AVG synapse is pruned in hermaphrodites, whereas the PHB > AVA synapse is pruned in males. Adapted from Barr et al. (2018). **(B)** Sexually dimorphic behavior influenced by differential gene expression in the AWA neuron. In hermaphrodites, ODR-10 expression is high in AWA, and thus feeding behavior is promoted. In males, ODR-10 expression depends on food availability; if food is plentiful, ODR-10 is downregulated, and thus mate searching is promoted. However, during starvation or the larval stages, ODR-10 is upregulated and feeding behavior is promoted. The expression ofSRD-1 in AWA also differs between the sexes, and this differential expression pattern contributes to the sex differences in response to the volatile sex pheromone; only males (with a high SRD-1 level) are attracted to the pheromone.

In adult hermaphrodites, the sensory neuron PHB synapses onto the interneuron AVA, comprising a neural circuit involved in chemorepulsive behavior (Hilliard et al., 2002). In adult males, however, the PHB > AVA connection is absent, and PHB rather forms synapses with the interneuron AVG and a subset of male-specific neurons, and these neuronal connections contribute to a circuit regulating male mating behavior (Oren-Suissa et al., 2016). Interestingly, this sexual dimorphism in neural connectivity is governed by a sex-specific synapse pruning mechanism. In larval stages, the PHB > AVA and PHB > AVG connections are both present in the two sexes. However, upon transition to adulthood, the PHB > AVA and PHB > AVG connections are specifically eliminated in males and hermaphrodites, respectively (Oren-Suissa et al., 2016; Figure 2A). These pruning events are thought to be mediated by a group of Double sex/MAB-3 domain transcription factors, including DMD-4, DMD-5, and DMD-11, all of which are downstream of TRA-1 (Oren-Suissa et al., 2016; Bayer et al., 2020a). These observations show the importance of the sex-determination pathway in generating sex-specific neural connectivity patterns, shedding light on how this regulation achieves such sex-specificity during development.

The sex-specific pruning or retention of the PHB > AVG connection appears to be mediated by the netrin signaling factors. UNC-6/netrin is expressed in the postsynaptic AVG during the larval stages of both males and hermaphrodites, but its expression is maintained after sexual maturation only in males. This dimorphic expression pattern of UNC-6 is directly regulated by TRA-1; in males, UNC-6 expression is relieved from TRA-1 repression and then mediates the retention of the PHB > AVG connection, whereas in hermaphrodites, UNC-6 is suppressed by TRA-1, and thus the connection is lost (Weinberg et al., 2018). Moreover, the netrin receptor UNC-40/DCC (Deleted in Colorectal Cancer) is active in the presynaptic PHB, and thus the PHB > AVG connection is retained in adult males. In adult hermaphrodites, UNC-40 is degraded by a ubiquitin ligase in the absence of UNC-6 in AVG, and the PHB > AVG synapse is consequently removed (Salzberg et al., 2020). These results suggest that sex-specific regulation of cell-surface proteins is crucial for dimorphic synapse formation, but whether other synaptic pairs showing sex differences use a similar mechanism or not awaits further research.

## Functional Differences

In addition to the above-mentioned sex differences, there is sexual dimorphism in neuronal function with no or minimal, if present, connectivity difference between the sexes. The known examples involve sex differences in gene expression and/or function of sex-shared sensory neurons in the head, including AWA, AWC, ASI, ASJ, ADF, ADL, and ASK, all of which contribute to the neural circuits for male mate searching or response to pheromones (White et al., 2007; Srinivasan et al., 2008; Jang et al., 2012; Jang and Bargmann, 2013; Ryan et al., 2014; Hilbert and Kim, 2017; Fagan et al., 2018; Luo and Portman, 2021). Since the sex differences in these neurons and their functional outcomes have been thoroughly reviewed elsewhere (Barr et al., 2018; Emmons, 2018), we will focus our discussion on AWA neurons, whose functions have been better studied than other neurons, as an example.

A sexually dimorphic function of the chemosensory AWA neurons involves a behavioral prioritization of feeding vs. mate searching. Under well-fed conditions, adult hermaphrodites tend to stay on bacterial food, whereas adult males leave food to search for mates (Lipton et al., 2004). This dimorphic behavior is in part mediated by a sex difference in the AWA expression of ODR-10, a chemoreceptor involved in food detection, which is in turn controlled by the genetic sex of the nervous system *via* TRA-1 (Lee and Portman, 2007; Ryan et al., 2014). Hermaphrodites highly express ODR-10 in AWA (due to active TRA-1), resulting in efficient food detection. In contrast, the low ODR-10 expression in males (due to inactive TRA-1) reduces food attraction and promotes mate searching (Figure 2B). Interestingly, it has been shown that ODR-10 expression in the male AWA and food attraction are induced during starvation or the larval stages (Ryan et al., 2014; Figure 2B). This condition-dependent modulation of ODR-10 expression is controlled by a non-cell-autonomous function of TGF-β and insulin signaling, which are thought to be downstream of or in parallel to TRA-1 (Lawson et al., 2020; Wexler et al., 2020).

Another dimorphic feature of AWA is a sex difference in response to the volatile sex pheromone, which is mediated by the expression of the chemoreceptor SRD-1 (Wan et al., 2019). The SRD-1 expression in the male AWA is high, resulting in attraction to the volatile sex pheromone, whereas hermaphrodites show no pheromone response or SRD-1 expression in AWA (Figure 2B). The dimorphic expression of SRD-1 is likely controlled by TRA-1 since changing the sexual identity of all the neurons from hermaphrodite to male resulted in SRD-1 upregulation in AWA (Wan et al., 2019).

Like the gene expression changes in AWA, there are multiple examples of sexually dimorphic gene expression in the sex-shared neurons. For instance, the expression of neurotransmitter identity genes has been monitored in both sexes to reveal neurotransmitter usage differences in some sex-shared neurons, including sensory neurons ADF, PHC, and PQR, interneurons AIM, PVN, and PVW, and motor neurons AS11 and PDB (Pereira et al., 2015 ; Serrano-Saiz et al., 2017b; Pereira et al., 2019). The functional consequences of these neurotransmitter identity changes are currently less understood.

## Concluding Remarks

In *C. elegans*, sexual dimorphism in neural anatomy and function harbors two components of the nervous system—sex-specific and sex-shared neurons. Even in the sex-shared neurons, there are remarkable differences in structure, synaptic connectivity, gene expression, and function between the sexes. These sex differences, in most cases, are governed by TRA-1, the master regulator of sexual identity, and its downstream transcription factors (e.g., DMD proteins) which in turn regulate the expression of target effector protein genes (e.g., neural cell-surface protein genes). However, even the regulation by TRA-1 is presumably not as simple as previously thought since recent studies have identified substantial variations in TRA-1 expression among sex-shared neurons depending on age, sex, or environmental conditions (Bayer et al., 2020b; Lawson et al., 2020). Nonetheless, as we have discussed, the initial probing of anatomical or behavioral sex differences (e.g., neurite pattern, synaptic connectivity, and response to environmental signals) and the following dissection of relevant molecular mechanisms have been fruitful, providing a good starting point for studying the sex differences in the nervous system. Therefore, comprehensive future studies on the nervous systems of model organisms encompassing connectomics, developmental genetics, and functional and behavioral analyses will help provide valuable insights into our understanding of how the nervous system differs between the sexes, how these sex differences become pronounced by environmental modulations, and perhaps why some human neurological disorders show a sex bias in their prevalence.

## Author Contributions

DK and BK developed the concept. Both have written and edited the text. All authors contributed to the article and approved the submitted version.

## Conflict of Interest

The authors declare that the research was conducted in the absence of any commercial or financial relationships that could be construed as a potential conflict of interest.

## Publisher’s Note

All claims expressed in this article are solely those of the authors and do not necessarily represent those of their affiliated organizations, or those of the publisher, the editors and the reviewers. Any product that may be evaluated in this article, or claim that may be made by its manufacturer, is not guaranteed or endorsed by the publisher.

## Footnotes

^1^ https://wormwiring.org
